# Bridging the gap in delirium management: a European comparison of geriatric practices

**DOI:** 10.1007/s41999-026-01527-6

**Published:** 2026-06-29

**Authors:** Alessandra Coin, Silvia Sturani, Christian Pozzi, Maela Caudal, Duygu Erbas Sacar, Massimiliano Fedecostante, Maria Cristina Ferrara, Antonio Cherubini, Alessandro Morandi, Gulistan Bahat, Giuseppe Bellelli, Suzanne Timmons

**Affiliations:** 1 Department of Medicine (DIMED), University of Padova, Padua, Italy; 2 Unit of Geriatrics, University Padova Hospital, Padua, Italy; 3 Centre of Competence on Ageing, University of Applied Sciences and Arts of Southern Switzerland (SUPSI), Lugano, Switzerland; 4 Altersmedizinisches Zentrum (Center of Geriatric Medicine), St. Marien-Hospital, Cologne, Germany; 5 Department of Internal Medicine, Division of Geriatrics, Kartal Dr. Lutfi Kirdar City Hospital, Istanbul, Türkiye; 6 Geriatria, Accettazione Geriatrica e Centro di Ricerca per l’Invecchiamento, IRCCS INRCA, Ancona, Italy; 7 School of Medicine and Surgery, University of Milano-Bicocca, Milan, Italy; 8 Department of Clinical and Molecular Sciences, Università Politecnica delle Marche, Ancona, Italy; 9 Department of Clinical and Experimental Science, University of Brescia, Brescia, Italy; 10 Azienda Speciale Cremona Solidale, Cremona, Italy; 11 Division of Geriatrics, Department of Internal Medicine, Istanbul Medical Faculty, Istanbul University, Capa, Istanbul, Türkiye; 12 Acute Geriatric Unit, IRCCS Foundation San Gerardo dei Tintori, Monza, Italy; 13 Centre for Gerontology and Rehabilitation, School of Medicine, University College Cork, Cork, Ireland

**Keywords:** Delirium, Geriatric Medicine, Older adults, European survey, Health service, Inequality

## Abstract

**Aim:**

To compare diagnostic and therapeutic approaches to delirium across European countries with well-established (WE) versus less-developed (LE) geriatric medicine (GM), to identify similarities, differences and potential gaps in care.

**Methods:**

A cross-sectional online survey was conducted among European geriatricians via the European Geriatric Medicine Society and professional networks from July 2023 to March 2024. Responses on delirium diagnosis and management were analyzed quantitatively, with comparisons between WE and LDGM countries.

**Results:**

Of 196 hospital-based respondents, 79.1% were from WEGM countries and 20.9% from LDGM countries. Structured delirium screening was significantly more common in WEGM nations, especially in geriatric (88.4% *vs* 43.9%, *p* < 0.001) and orthopedic wards. Non-pharmacological strategies—such as hydration support (76.8% *vs* 52.5%, *p* = 0.002) and family involvement (81.5% *vs* 60.4%, *p* = 0.005)—were also more frequently used. Conversely, physical restraints were more commonly employed in LDGM countries (70.7% *vs* 41.4%, *p* < 0.001), as were pharmacological treatments, such as trazodone and quetiapine.

**Conclusion:**

Marked differences exist in delirium management across Europe, which may be linked at least in part to GM development, although confounding factors may exist, and causality cannot be assumed. WEGM countries appear to prioritize structured screening and non-pharmacological interventions, whereas LDGM countries rely more on pharmacological treatments. These findings highlight the need for standardized European guidelines to ensure equitable delirium care.

**Supplementary Information:**

The online version contains supplementary material available at https://doi.org/10.1007/s41999-026-01527-6.

## Key summary points

**Aim:**

To compare diagnostic and therapeutic approaches to delirium across European countries with well-established versus less-developed Geriatric Medicine (GM), to identify similarities, differences, and potential gaps in care.

**Findings:**

Countries with well-established GM more frequently reported structured delirium screening, established management protocols, and the use of non-pharmacological interventions. In contrast, countries with less-developed GM showed higher reliance on pharmacological treatments and physical restraints.

**Message:**

Marked differences in delirium management persist across Europe and appear to be associated with the level of development of Geriatric Medicine. These findings highlight the need for harmonized European guidelines to promote equitable, evidence-based delirium care.

**Supplementary Information:**

The online version contains supplementary material available at https://doi.org/10.1007/s41999-026-01527-6.

## Introduction

Delirium is an acute geriatric syndrome affecting attention, cognition, and psychomotor activity, with a fluctuating course, which occurs primarily in older adults [1]. It often arises due to underlying medical conditions [2] and is strongly associated with frailty, particularly in acute [3–5] and long-term care settings.

Prevalence varies by setting: 1–2% in community-dwelling older adults, rising to 14–24% in hospitalized patients and up to 80% in critical care [6–9]. Often underrecognized, delirium leads to increased disability, mortality, institutionalization, dementia risk, and substantial healthcare costs [10–12].

Accurate investigation requires cognitive assessment and a thorough history. National guidelines recommend routine delirium screening using validated tools, with the Confusion Assessment Method (CAM) and the 4 ‘A’s Test (4AT) being the most widely used [13–17]. Despite recommendations to screen all older emergency admissions for dementia and delirium [14], no consensus exists on standardized evaluation methods [18].

Non-pharmacological interventions effectively reduce delirium risk [14, 19] and growing evidence supports multimodal, multidisciplinary approaches as highly effective [20–23].

In addition to delirium prevention, standardized treatment guidelines are essential. Currently, there are no European-specific guidelines for delirium management. Addressing modifiable causes identified during evaluation is critically important, as even minor interventions can have a significant impact [6, 24].

No universally accepted pharmacological treatment exists. The use of antipsychotics and sedatives is not recommended, except in cases of hyperactive delirium, for short durations, and only when the patient is in severe distress or at risk of disrupting life-sustaining treatments. Nonetheless, such medications are still commonly used in clinical practice [25]. Low doses of haloperidol are considered as first-line agent in the symptomatic management of delirium episodes [26]. These data underscore the importance of prioritizing non-pharmacological strategies and individualized risk assessment in the prevention and management of delirium in older adults [27, 28]. Variability in both pharmacological and non-pharmacological approaches complicates standardization, further influenced by cultural differences across countries.

Moreover, although several national and international guidelines exist, there is currently no unified European framework specifically addressing delirium management, which may contribute to heterogeneity in clinical practice across countries [24, 29].

Considering the variability in delirium management and the lack of homogeneity in following guidelines across European countries, another relevant aspect to address is the potential variability between nations where Geriatric Medicine is an established medical specialty and those where it is not yet formally recognized or integrated into postgraduate medical education. This study compares diagnostic and therapeutic approaches for delirium between countries with well-established (WE) versus less-developed (LD) Geriatric Medicine (GM) medical specialty, aiming to outline the similarities and differences, and thereby contribute to the recognition of the need for, and the development of, standardized guideline measures across Europe [29].

## Methods

The survey was created and revised throughout the members of the research team, who are all geriatricians. Four of the authors considered to be experts in the field (GB, AC, ST, AM) gave feedback on the face and content validity of each single question and on the overall questionnaire.

After discussion in the working group and with the Ethical Committees of the University of Padova and the University of Dublin, it was decided that it was not necessary to have an ethical consent due to the nature of the survey, not involving patients, but only asking opinion of physicians in an anonymous way.

The survey was structured in 4 sections (Supplementary file 1).

At the beginning of the survey, participants were informed about the aim of this survey, an estimation of how much time was needed to fulfill it, and that their participation accepted the conditions. Participation was voluntary, and completion of the survey was considered implied informed consent. Responses reflect clinicians’ reported perceptions of practices within their unit or hospital rather than externally verified institutional policies. The survey was mainly multiple-choice questions, but the participant was given the possibility to give more detailed information about some questions. No patients’ data were acquired and the answers were anonymous. In the first section, a brief overview of participant characteristics was requested, while respecting their anonymity. In the second section, the participant was requested to answer questions about the different wards present in his/her hospital, the existing delirium diagnostic pathway and delirium-frailty pathway in those wards. Specifically, the considered wards were: geriatric, ICU, emergency, orthopedic, medical ward, surgery ward, rehabilitation and long-term facilities. However, the answers concerning rehabilitation and long-term facilities were not considered for the present manuscript (Supplementary Table 1). The aim in this section was to determine if a structured pathway to identify delirium in the different wards exist.

In the third section, participants were asked about the usual treatment of delirium in their hospital. Here, there was no difference made between the wards. The use of non-pharmacological methods was investigated by multiple-choice questions. For each positive answer, a percentage for the use of this method among patients was asked (< 25%; 25–50%; 50–75%; > 75%). Finally, pharmacological methods were examined. For each medication, the participant could specify the usual dose and the type of administration. In this section, the aim was to identify the usual methods used to treat delirium with pharmacological as well as non-pharmacological methods.

The survey was conducted among geriatricians who have an affiliation to the EuGMS (European Geriatric Medicine Society) or were contacted through professional networks and are working in Europe. It was presented as a Google form in English. It was distributed by email through the EuGMS and cascaded in a snowball approach via professional networks. The authors also distributed the questionnaire through their respective national or in-country professional networks.

The survey was launched in July 2023 and closed in March 2024. Participants were asked to provide answers about their own wards and the other wards of their hospital.

### Analysis

After excluding answers from countries outside of Europe and incomplete surveys, responses obtained by participants were divided into two groups according to the presence of a recognized primary specialty in geriatric medicine (GM) in the represented countries [29]: well-established GM (Belgium, Czech Republic, Denmark, Finland, France, Germany, Iceland, Ireland, Italy, Lithuania, Luxemburg, Malta, Norway, Poland, Romania, Spain, Sweden, The Netherlands, United Kingdom) and less-developed GM (Belarus, Greece, Portugal, Slovenia, Switzerland, Turkiye). This classification is based on a previously published European framework reflecting the recognition of geriatric medicine as a primary specialty and its integration into postgraduate training pathways and dedicated geriatric services. Quantitative survey data were analyzed statistically. Nominal data were reported as numbers and percentages. The Chi-Square test was conducted to examine differences between countries. Statistical analyses were performed using SPSS 26.0 (IBM Corp.) It was hypothesized that countries with well-established GM had a better management of delirium, e.g. higher rate of delirium diagnosis, as well as a more frequent non-pharmacological treatment.

## Results

In total, 238 physicians accessed and completed the survey. Thirteen responses coming from countries outside Europe and 6 incomplete ones were excluded. Because of the aim of the study, 23 responses from a non-hospital setting were also excluded, giving a final sample of 196 participants. According to the pre-defined classification, 41 (20.9%) participants were from countries with less-established GM while 155 (79.1%) were from countries with well-established GM (Table 1).

**Table 1:** List of survey participants countries

	*N* (%)
**Less-developed GM (*N* = 41)**	
Belarus	1 (2.4)
Greece	4 (9.8)
Portugal	10 (24.4)
Slovenia	1 (2.4)
Switzerland	12 (29.3)
Turkey	13 (31.7)
**Well-established GM (*N* = 155)**	
Belgium	14 (9)
Czech Republic	2 (1.3)
Denmark	12 (7.7)
Finland	2 (1.3)
France	16 (10.3)
Germany	9 (5.8)
Iceland	1 (0.6)
Ireland	6 (3.9)
Italy	17 (11)
Lithuania	1 (0.6)
Luxemburg	1 (0.6)
Malta	2 (1.3)
Norway	12 (7.7)
Poland	8 (5.2)
Romania	2 (1.3)
Spain	19 (12.3)
Sweden	4 (2.6)
The Netherlands	10 (6.5)
United Kingdom	17 (11)

General characteristics of the structures where participants work, the prevalence of screening tools, and the prevalence of protocols and pathways for delirium and frailty according to different medical or surgical wards are reported in Table 2. Participants were mainly from University or University-related hospitals (73.0%), with an equal distribution of the hospital size from < 250 to > 1000 beds. A geriatric ward was present in 79.1% of hospitals, with a significant higher prevalence in countries with well-established GM (88.4% vs. 43.9%; *p* < 0.001). The use of a screening tool was more frequently reported in well-established GM countries both in geriatric wards (71.6% vs. 34.1%; *p* < 0.001) and in orthopedic wards (49.0% vs. 19.5%; *p* = 0.002). Likewise, the presence of a protocol for patients with delirium was more common in well-established GM countries in geriatric wards (52.3% vs. 29.3%; *p* < 0.001) and orthopedic wards (40.6% vs. 17.1%; *p* = 0.007).

**Table 2:** Survey: characteristics of the hospital facilities where participants work

Variables:	Less-developed GM (*N* = 41)	Well-established GM (*N* = 155)	*P*-value
**Type of structure**	Number (percent)		< 0.001
Community hospital	11 (26.8)	29 (18.7)	
Private hospital	8 (19.5)	5 (3.2)	
University hospital	16 (39)	90 (58.1)	
University-related/affiliated hospital	6 (14.6)	31 (20)	
**Size of the hospital (beds)**			0.712
< 250	13 (31.7)	37 (23.9)	
250–500	8 (19.5)	39 (25.2)	
500–1000	11 (26.8)	47 (30.3)	
> 1000	9 (22)	32 (20.6)	
**Presence of a geriatric ward**	18 (43.9)	137 (88.4)	** < 0.001**
Screening tool for delirium	14 (34.1)	111 (71.6)	** < 0.001**
Protocol for patients with delirium	12 (29.3)	81 (52.3)	** < 0.001**
Pathway including frailty assessment	8 (19.5)	52 (33.5)	0.066
Pathway including frailty bundle care plan	8 (19.5)	48 (31)	0.073
Clear link with frailty bundle care plan	4 (9.8)	42 (27.1)	**0.017**
Referral for follow-up of cognition	9 (22)	56 (36.1)	0.065
**Presence of an Emergency Room**	35 (85.4)	134 (86.5)	0.858
Screening tool for delirium	10 (24.4)	74 (47.7)	**0.034**
Protocol for patients with delirium	7 (17.1)	52 (33.5)	0.229
Pathway including frailty assessment	4 (9.8)	36 (23.2)	0.237
Pathway including frailty bundle care plan	5 (12.2)	29 (18.7)	0.785
Clear link with frailty bundle care plan	3 (7.3)	25 (16.1)	0.464
Referral for follow-up of cognition	4 (9.8)	27 (17.4)	0.690
**Presence of an ICU**	36 (87.8)	136 (87.7)	0.991
Screening tool for delirium	19 (46.3)	67 (43.2)	0.826
Protocol for patients with delirium	11 (26.8)	38 (24.5)	0.240
Pathway including frailty assessment	4 (9.8)	14 (9)	0.676
Pathway including frailty bundle care plan	5 (12.2)	13 (8.4)	0.082
Clear link with frailty bundle care plan	5 (12.2)	12 (7.7)	0.178
Referral for follow-up of cognition	6 (14.6)	11 (7.1)	0.364
**Presence of a Medical ward**	36 (87.8)	144 (92.9)	0.289
Screening tool for delirium	16 (39)	76 (49)	0.510
Protocol for patients with delirium	14 (34.1)	59 (38.1)	0.174
Pathway including frailty assessment	6 (14.6)	25 (16.1)	0.285
Pathway including frailty bundle care plan	7 (17.1)	27 (17.4)	0.242
Clear link with frailty bundle care plan	5 (12.2)	26 (16.8)	0.619
Referral for follow-up of cognition	10 (24.4)	26 (16.8)	0.169
**Presence of a Surgical ward**	33 (80.5)	139 (89.7)	0.110
Screening tool for delirium	9 (22)	55 (35.5)	0.193
Protocol for patients with delirium	7 (17.1)	42 (27.1)	0.195
Pathway including frailty assessment	3 (7.3)	21 (13.5)	0.402
Pathway including frailty bundle care plan	3 (7.3)	22 (14.2)	0.532
Clear link with frailty bundle care plan	3 (7.3)	17 (11)	0.307
Referral for follow-up of cognition	4 (9.8)	19 (12.3)	0.722
**Presence of an Orthopedic ward**	30 (73.2)	130 (83.9)	0.116
Screening tool for delirium	8 (19.5)	76 (49)	**0.002**
Protocol for patients with delirium	7 (17.1)	63 (40.6)	**0.007**
Pathway including frailty assessment	3 (7.3)	43 (27.7)	**0.047**
Pathway including frailty bundle care plan	3 (7.3)	40 (25.8)	0.057
Clear link with frailty bundle care plan	3 (7.3)	37 (23.9)	0.086
Referral for follow-up of cognition	4 (9.8)	40 (25.8)	0.172

Statistically significant *p*-values are highlighted in bold to distinguish them from non-significant values

**Fig. 1:**
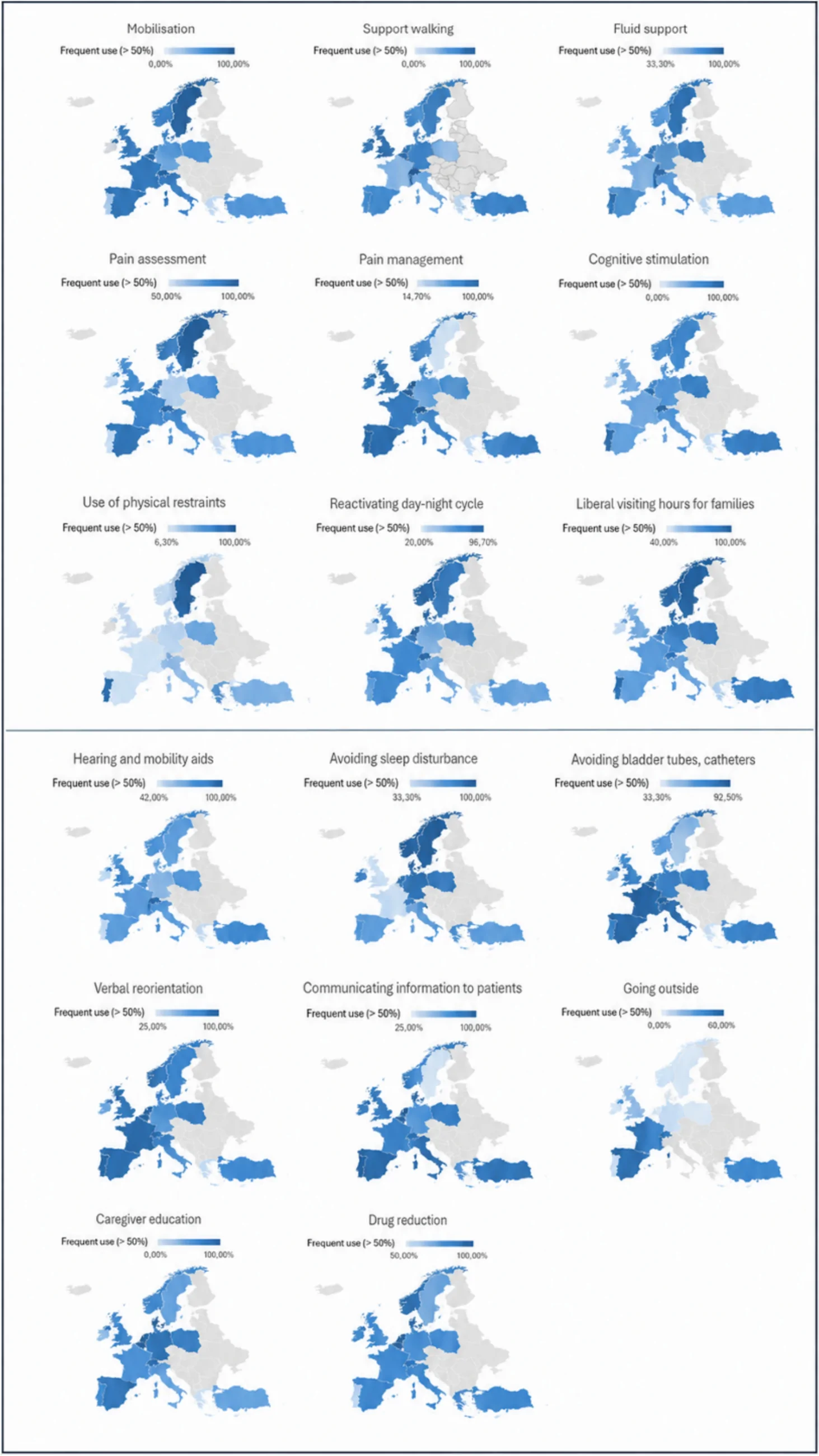
Geographical distribution of non-pharmacological strategies (> or < 50%) by country—blue gradient map. Belarus, Czech Republic, Finland, Iceland, Lithuania, Luxembourg, Malta, Romania, and Slovenia are not represented in the maps because they had fewer than 4 responses

The different non-pharmacological and pharmacological management strategies for delirium are shown in Tables 3 and 4. Countries with a well-established GM discipline showed a significantly higher prevalence of non-pharmacological approaches, such as support to increase fluid intake, open or liberal visiting times for families, avoidance of unnecessary bladder tubes or catheters, reduction of drugs potentially associated with delirium, and more frequent (> 50% of times) use of patient mobilization. There was also a significantly less frequent use of physical restraints in countries with well-developed GM compared to those with less-developed GM. Figure 1 shows the geographical distribution of countries based on whether non-pharmacological strategies are reported to be used in more or less than 50% of cases. The responses are visualized using a gradient of blue shades.

**Table 3:** The table shows responses on the use of non-pharmacological intervention strategies, including the number (and percentage) of affirmative responses for each strategy and those with a utilization rate > 50%

Variables	Less-developed GM (*N* = 41)	Well-established GM (*N* = 155)	*P*-value
Mobilization	35 (85.4)	148 (95.5)	0.061
of which, use > 50%	18 (51.4)	107 (72.3)	**0.017**
Support to increase mobility	20 (48.8)	92 (59.4)	0.243
of which, use > 50%	13 (65)	54 (58.7)	0.602
Pain assessment	41 (100)	146 (94.2)	0.196
of which, use > 50%	33 (80.5)	121 (82.9)	0.723
Pain management	29 (70.7)	112 (72.3)	0.734
of which, use > 50%	21 (72.4)	79 (70.5)	0.843
Physical restraints	31 (75.6)	105 (67.7)	0.448
of which, use > 50%	12 (38.7)	16 (15.2)	**0.005**
Reactivate day and night cycle	35 (85.4)	130 (83.9)	0.871
of which, use > 50%	23 (65.7)	89 (68.5)	0.757
Support to increase fluid intake	30 (73.2)	130 (83.9)	**0.021**
of which, use > 50%	25 (80)	96 (73.8)	0.483
Provision of hearing and mobility aids	33 (80.5)	132 (85.2)	0.174
of which, use > 50%	23 (69.7)	101 (76.5)	0.418
Cognitive stimulation	28 (68.3)	105 (67.7)	0.651
of which, use > 50%	18 (64.3)	52 (49.5)	0.165
Verbal reorientation	39 (95.1)	145 (93.5)	0.941
of which, use > 50%	31 (79.5)	125 (86.2)	0.300
Open or liberal visiting times for families	35 (85.4)	144 (92.9)	**0.040**
of which, use > 50%	28 (80)	117 (81.3)	0.866
Caregiver education for proactive and informed caregiving	29 (70.7)	117 (75.5)	0.171
of which, use > 50%	17 (58.6)	77 (65.8)	0.469
Transferring to specific delirium rooms	3 (7.3)	25 (16.1)	0.148
of which, use > 50%	0 (0)	11 (44)	0.140
Non-disturbed sleep	27 (65.9)	96 (61.9)	0.575
of which, use > 50%	19 (70.4)	65 (67.7)	0.793
Avoidance of unnecessary bladder tubes or catheters	36 (87.8)	145 (93.5)	**0.045**
of which, use > 50%	27 (75)	114 (78.6)	0.639
Sharing or communicating patient information about delirium	38 (92.7)	145 (93.5)	0.608
of which, use > 50%	33 (86.8)	124 (85.5)	0.835
Ground-leveled beds	15 (36.6)	71 (45.8)	0.238
of which, use > 50%	5 (33.3)	26 (36.6)	0.810
Group activities for patients	14 (34.1)	49 (31.6)	0.741
of which, use > 50%	6 (42.9)	13 (26.5)	0.240
Animal assisted therapy	3 (7.3)	12 (7.7)	0.919
of which, use > 50%	1 (33.3)	2 (16.7)	0.519
Going outside the unit ward	15 (36.6)	35 (22.6)	0.083
of which, use > 50%	4 (26.7)	6 (17.1)	0.440
Reduction of potentially deliriogenic drugs	37 (90.2)	149 (96.1)	**0.041**
of which, use > 50%	29 (78.4)	128 (85.9)	0.259

Statistically significant *p*-values are highlighted in bold to distinguish them from non-significant values

**Table 4:** The table shows responses on the use of pharmacological intervention strategies, including the number (and percentage) of affirmative responses for each strategy and those with a utilization rate > 50%

Variables	Less-established GM (*N* = 41)	Well-established GM (*N* = 155)	*P*-value
Delirious patients receiving pharmacological intervention	41 (100)	152 (98.1)	0.369
of which, use > 50%	29 (70.7)	63 (41.4)	** < 0.001**
Haloperidol	32 (78)	122 (78.7)	0.927
Clonidine	1 (2.4)	2 (1.3)	0.594
Promazine	0 (0)	7 (4.5)	0.166
Trazodone	23 (56.1)	43 (27.7)	** < 0.001**
Melperone	1 (2.4)	10 (6.5)	0.321
Risperidone	21 (51.2)	95 (61.3)	0.243
Olanzapine	16 (39)	49 (31.6)	0.370
Lorazepam	12 (29.3)	54 (34.8)	0.502
Dexmedetomidine	1 (2.4)	6 (3.9)	0.660
Diazepam	4 (9.8)	16 (10.3)	0.915
Quetiapine	39 (95.1)	103 (66.5)	** < 0.001**
Midazolam	4 (9.8)	32 (20.6)	0.109
Distraneurin	4 (9.8)	10 (6.5)	0.465
Melatonin	17 (41.5)	51 (32.9)	0.306
Beta-blocker	1 (2.4)	6 (3.9)	0.660
Pipamperone	2 (4.9)	5 (3.2)	0.612
Levodopa	1 (2.4)	5 (3.2)	0.795
Phenobarbital	1 (2.4)	0 (0)	0.051

Statistically significant *p*-values are highlighted in bold to distinguish them from non-significant values

**Fig. 2:**
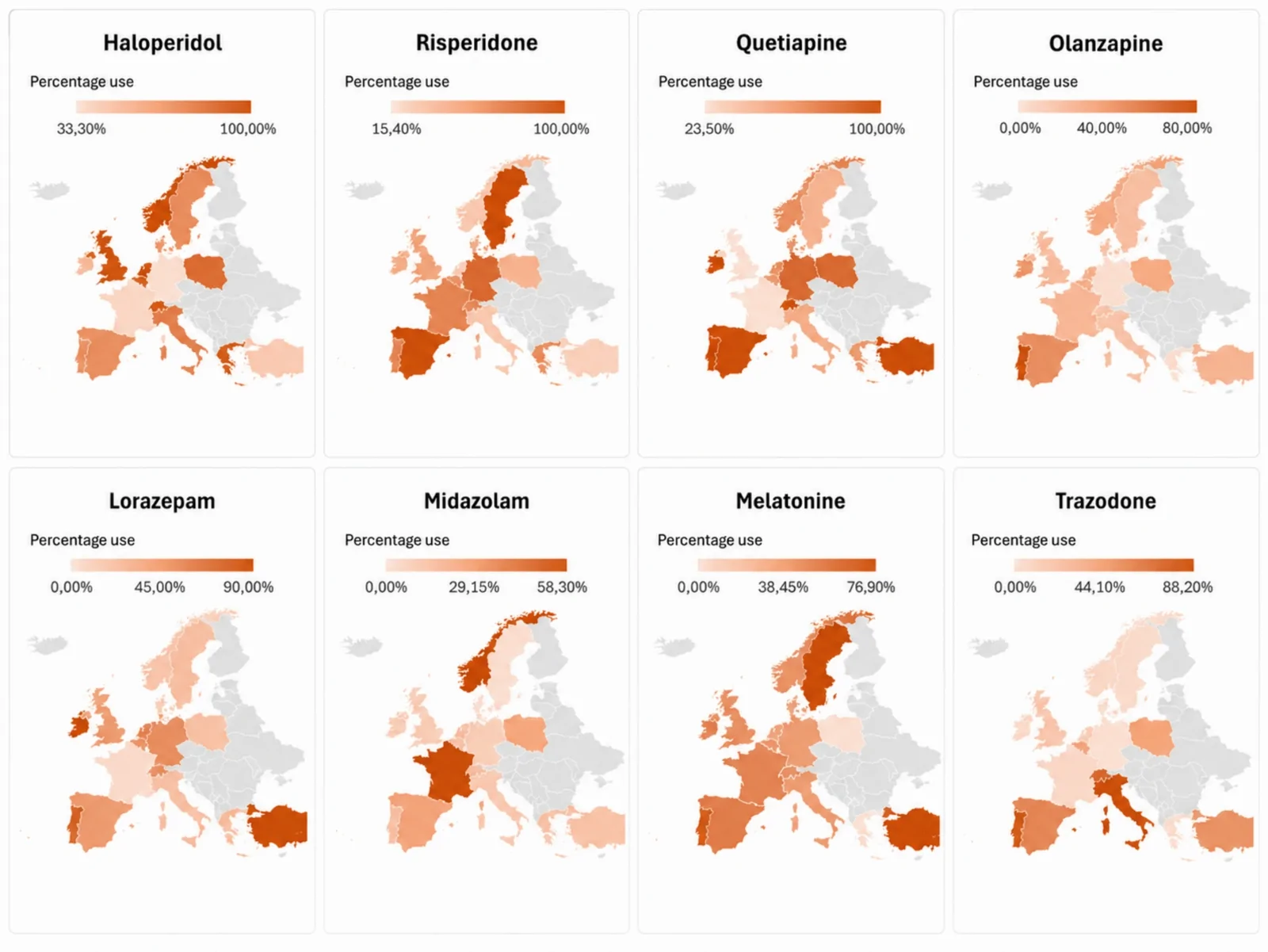
Geographical distribution of medication usage percentages—orange gradient map. Belarus, Czech Republic, Finland, Iceland, Lithuania, Luxembourg, Malta, Romania, and Slovenia are not represented in the maps because they had fewer than 4 responses

Pharmacological approaches were significantly less often used in countries with a well-established GM (41.4% vs. 70.7%; *p* < 0.001). Among different drugs, trazodone and quetiapine were significantly less used in countries with well-established GM (27.7% vs. 56.1% and 66.5% vs. 95.1%, respectively; all *p* < 0.001). Figure 2 illustrates the geographical distribution of medication usage percentages (the gradient of orange shades reflects the proportion of respondents indicating the use of each medication).

## Discussion

The results of this survey highlight significant differences in the diagnosis and management of delirium across European countries, supporting the hypothesis that differences in the level of development of geriatric medicine may influence clinical practice. The presence of a dedicated geriatric ward was significantly more common in well-established GM countries, which likely reflects a stronger institutional focus on geriatric care. This structural difference may contribute to the observed variations in screening and management practices.

A key finding was the higher prevalence of routine delirium screening and established management protocols in well-established GM countries. The use of validated screening tools, particularly in geriatric and orthopedic wards, was significantly more frequent in these countries. This aligns with existing literature, which emphasizes that early identification of delirium is crucial for improving patient outcomes [26]. Screening facilitates timely interventions that can reduce the severity and duration of delirium episodes, potentially decreasing associated morbidity and mortality.

The survey also revealed notable differences in non-pharmacological management strategies. Countries with well-established GM were more likely to implement evidence-based interventions, such as mobilization, hydration support, liberal family visiting policies, and avoidance of potentially deliriogenic medications. These findings are consistent with research indicating that multimodal, non-pharmacological interventions are the most effective strategies for preventing and managing delirium [30, 31].

One notable result concerns the use of physical restraints. The survey revealed significant differences between countries, with well-established GM countries demonstrating a significantly lower prevalence of restraint use. Despite strong recommendations against their routine use due to the risk of severe complications, such as increased agitation, functional decline, pressure ulcers, and thromboembolic events [32], physical restraints were still commonly used across all surveyed countries. In particular, their overall utilization was higher than what is typically reported in the literature, which suggests that restraints continue to be a widespread, but suboptimal practice in delirium management [33]. The paradoxical effect of physical restraints, whereby they may worsen delirium symptoms by increasing stress and limiting mobility, has been well-documented [34]. The reduced use of restraints in well-established GM countries suggests greater use of alternative management strategies, such as increased supervision, environmental modifications, and caregiver education. These findings reinforce the need for continued efforts to discourage restraint use and promote evidence-based, patient-centered delirium management.

The observed differences in the prevalence of neuroleptic and sedative use in less-established GM countries indirectly suggest a lower reliance on non-pharmacological approaches, which are more commonly implemented in WEGM countries. However, these findings should be interpreted with caution, as the questionnaire did not distinguish the specific clinical indication for each medication (e.g., delirium treatment, agitation, sleep disturbances, or other conditions) and, therefore, may not fully reflect delirium-specific prescribing practices. This highlights the critical need for targeted education and training in these regions to discourage pharmacological interventions, except in cases of severely agitated hyperactive delirium, and only after non-pharmacological strategies have been exhausted or when the patient is experiencing significant distress or is at risk of interrupting life-sustaining treatments. Surveys such as this are also valuable for demonstrating the feasibility of alternative approaches in contexts where geriatric care models are less established [29, 34–36].

These findings underscore the need for standardized European guidelines to harmonize delirium management. The observed discrepancies highlight the potential for improved patient outcomes through wider adoption of structured screening, evidence-based non-pharmacological interventions, and judicious use of pharmacological treatments. Developing consensus-based protocols across Europe could help ensure equitable and high-quality care for older adults at risk of delirium, regardless of geographic location or healthcare system structure.

This study has several limitations. First, the survey captured self-reported information from clinicians regarding practices in their unit or hospital and, therefore, reflects perceived practice rather than externally verified institutional policies. Second, responses were analyzed at the individual level without accounting for potential clustering within countries, as clinical practices may be influenced by national healthcare systems, training structures, and local guidelines. Third, the number of respondents varied substantially across countries, with some countries represented by only a few participants; therefore, country-level findings and visual representations should be interpreted with caution. Fourth, participation was voluntary and distributed through professional networks, introducing the possibility of selection bias; moreover, the survey was conducted in English only, which may also have introduced a selection bias related to language proficiency. Finally, the questionnaire did not specify the clinical indication for pharmacological treatments, limiting the interpretation of these data.

## Conclusion

This survey highlights relevant differences in the diagnosis and management of delirium across European countries, reflecting variability in geriatric practices and healthcare organization. Countries with more established geriatric services report more frequent use of routine screening, structured management protocols, and non-pharmacological interventions, together with lower use of physical restraints. In contrast, in settings where geriatric medicine is less developed, delirium care appears more often based on pharmacological or restrictive approaches. Although the observational nature of this study does not allow causal interpretations, the findings suggest the presence of a gap in delirium care across Europe. Query Efforts aimed at greater alignment of diagnostic and management practices may help reduce this gap and promote more consistent delirium care for older hospitalized patients.

## Supplementary Information

Below is the link to the electronic supplementary material.

Supplementary file1 (DOCX 15 KB)

Supplementary file2 (PDF 1177 KB)

## Data Availability

The data generated from this survey are available from the EuGMS Special Interest Group (SIG) on Delirium upon reasonable request and subject to approval by the SIG.

## References

[1] American Psychiatric Association, American Psychiatric Association Task Force on DSM-IV (1994) Diagnostic and statistical manual of mental disorders: DSM-IV, 4th edn. American Psychiatric Association, Washington, DC

[2] Evensen S, Saltvedt I, Lydersen S, Wyller TB, Taraldsen K, Sletvold O (2019) Delirium motor subtypes and prognosis in hospitalized geriatric patients – a prospective observational study. J Psychosom Res 122:24–28 31126407 10.1016/j.jpsychores.2019.04.020

[3] Geriatric Medicine Research Collaborative (2023) Increasing frailty is associated with higher prevalence and reduced recognition of delirium in older hospitalised inpatients: results of a multi-centre study. Eur Geriatr Med 14(2):325–332 36696045 10.1007/s41999-022-00737-y PMC10113325

[4] Cechinel C, Lenardt MH, Rodrigues JAM, Binotto MA, Aristides MM, Kraus R (2022) Frailty and delirium in hospitalized older adults: a systematic review with meta-analysis. Rev Lat Am Enfermagem 17(30):e3687 10.1590/1518-8345.6120.3687 PMC9580989 36287400

[5] Persico I, Cesari M, Morandi A, Haas J, Mazzola P, Zambon A, Annoni G, Bellelli G (2018) Frailty and delirium in older adults: a systematic review and meta-analysis of the literature. J Am Geriatr Soc 66(10):2022–2030. 10.1111/jgs.15503 30238970

[6] Inouye SK, Westendorp RGJ, Saczynski JS (2014) Delirium in elderly people. Lancet 383:911–922 23992774 10.1016/S0140-6736(13)60688-1 PMC4120864

[7] Bellelli G, Morandi A, Di Santo SG et al (2016) “Delirium Day”: a nationwide point prevalence study of delirium in older hospitalized patients using an easy standardized diagnostic tool. BMC Med 14:106 27430902 10.1186/s12916-016-0649-8 PMC4950237

[8] Han JH, Wilson A, Ely EW (2010) Delirium in the older emergency department patient: a quiet epidemic. Emerg Med Clin N Am 28(3):611–631 10.1016/j.emc.2010.03.005 PMC3708798 20709246

[9] Fuchs S, Bode L, Ernst J et al (2020) Delirium in elderly patients: prospective prevalence across hospital services. Gen Hosp Psychiatry 67:19–25 32911278 10.1016/j.genhosppsych.2020.08.010

[10] Davis DH, Muniz-Terrera G, Keage HA et al (2017) Association of delirium with cognitive decline in late life: a neuropathologicstudy of 3 population-based cohort studies. JAMA Psychiat 74:244–251 10.1001/jamapsychiatry.2016.3423 PMC6037291 28114436

[11] Wilson JE, Mart MF, Cunningham C et al (2020) Delirium. Nat Rev Dis Primers 6:90 33184265 10.1038/s41572-020-00223-4 PMC9012267

[12] Bellelli G, Brathwaite JS, Mazzola P (2021) Delirium: a marker of vulnerability in older people. Front Aging Neurosci 30(13):626127 10.3389/fnagi.2021.626127 PMC8119654 33994990

[13] American Geriatrics Society expert panel on postoperative delirium in older a (2015) American geriatrics society abstracted clinical practice guideline for postoperative delirium in older adults. J Am Geriatr Soc 63(1):142–150 25495432 10.1111/jgs.13281 PMC5901697

[14] (2023) Delirium: prevention, diagnosis and management in hospital and long-term care. London: National Institute for Health and Care Excellence (NICE) 31971702

[15] Han JH, Schnelle JF, Ely EW (2014) The relationship between a chief complaint of “altered mental status” and delirium in older emergency department patients. Acad Emerg Med 21:937–940 25154589 10.1111/acem.12436 PMC4150739

[16] Inouye SK, van Dyck CH, Alessi CA, Balkin S, Siegal AP, Horwitz RI (1990) Clarifying confusion: the confusion assessment method. a new method for detection of delirium. Ann Intern Med 113:941–948 2240918 10.7326/0003-4819-113-12-941

[17] Bellelli G, Morandi A, Davis DH, Mazzola P, Turco R, Gentile S, Ryan T, Cash H, Guerini F, Torpilliesi T et al (2014) Validation of the 4AT, a new instrument for rapid delirium screening: a study in 234 hospitalised older people. Age Ageing 43:496–502 24590568 10.1093/ageing/afu021 PMC4066613

[18] Zucchelli A, Apuzzo R, Paolillo C, Prestipino V, De Bianchi S, Romanelli G, Padovani A, Marengoni A, Bellelli G (2021) Development and validation of a delirium risk assessment tool in older patients admitted to the emergency department observation unit. Aging Clin Exp Res 33(10):2753–2758 33565046 10.1007/s40520-021-01792-4 PMC8531045

[19] Golubovic J, Neerland BE, Aune D, Baker FA (2022) Music interventions and delirium in adults: a systematic literature review and meta-analysis. Brain Sci 12(5):568 35624955 10.3390/brainsci12050568 PMC9138821

[20] Chalfont G, Milligan C, Simpson J (2020) A mixed methods systematic review of multimodal non-pharmacological interventions to improve cognition for people with dementia. Dement Lond Engl 19(4):1086–1130 10.1177/1471301218795289 PMC7180318 30193536

[21] Cuevas-Lara C et al (2019) Effectiveness of occupational therapy interventions in acute geriatric wards: a systematic review. Maturitas 127:43–50 31351519 10.1016/j.maturitas.2019.06.005

[22] Salvi F, Young J, Lucarelli M, Aquilano A, Luzi R, Dell’Aquila G, Cherubini A (2020) Non-pharmacological approaches in the prevention of delirium. Eur Geriatr Med 11(1):71–81 32297241 10.1007/s41999-019-00260-7

[23] Sonia M, Kaur S, Kothari N (2025) Prevention of delirium in the intensive care unit through nonpharmacological interventions: an umbrella review. Indian J Crit Care Med 29(1):75–83 39802256 10.5005/jp-journals-10071-24884 PMC11719550

[24] Marcantonio ER (2011) In the clinic: delirium. Ann Intern Med 154(11):ITC6–ITC1 21646553 10.7326/0003-4819-154-11-201106070-01006

[25] Tomlinson EJ, Schnitker LM, Casey PA (2024) Exploring antipsychotic use for delirium management in adults in hospital, sub-acute rehabilitation and aged care settings: a systematic literature review. Drugs Aging 41(6):455–486 38856874 10.1007/s40266-024-01122-z PMC11193698

[26] Campbell N, Boustani MA, Ayub A, Fox GC, Munger SL, Ott C, Guzman O, Farber M, Ademuyiwa A, Singh R (2009) Pharmacological management of delirium in hospitalized adults–a systematic evidence review. J Gen Intern Med 24(7):848–853 19424763 10.1007/s11606-009-0996-7 PMC2695535

[27] Burry L, Mehta S, Perreault MM, Luxenberg JS, Siddiqi N, Hutton B, Fergusson DA, Bell C, Rose L (2018) Antipsychotics for treatment of delirium in hospitalised non-ICU patients. Cochrane Database Syst Rev 6:CD005594 29920656 10.1002/14651858.CD005594.pub3 PMC6513380

[28] Soiza RL, Myint PK (2019) The Scottish intercollegiate guidelines network (SIGN) 157: guidelines on risk reduction and management of delirium. Medicina (Kaunas) 55(8):491 31443314 10.3390/medicina55080491 PMC6722546

[29] Stuck AE, Masud T (2022) Health care for older adults in Europe: how has it evolved and what are the challenges? Age Ageing 51(12):afac287 36529998 10.1093/ageing/afac287 PMC9760902

[30] Tahir TA, Eeles E, Karapareddy V, Muthuvelu P, Chapple S, Phillips B, Adyemo T, Farewell D, Bisson JI (2010) A randomized controlled trial of quetiapine versus placebo in the treatment of delirium. J Psychosom Res 69(5):485–490 20955868 10.1016/j.jpsychores.2010.05.006

[31] Grover S, Mahajan S, Chakrabarti S, Avasthi A (2016) Comparative effectiveness of quetiapine and haloperidol in delirium: a single blind randomized controlled study. World J Psychiatry 6(3):365–371 27679777 10.5498/wjp.v6.i3.365 PMC5031938

[32] Hatta K, Kishi Y, Wada K, Odawara T, Takeuchi T, Shiganami T, Tsuchida K, Oshima Y, Uchimura N, Akaho R, Watanabe A, Taira T, Nishimura K, Hashimoto N, Usui C, Nakamura H (2014) Antipsychotics for delirium in the general hospital setting in consecutive 2453 inpatients: a prospective observational study. Int J Geriatr Psychiatry 29(3):253–262 23801358 10.1002/gps.3999 PMC4229063

[33] Cui N, Qiu R, Zhang Y, Chen D, Zhang H, Rao H, Jin J (2021) Why are physical restraints still in use? a qualitative descriptive study from Chinese critical care clinicians perspectives. BMJ Open 11(11):e055073 34732505 10.1136/bmjopen-2021-055073 PMC8572407

[34] Berger S, Grzonka P, Amacher SA, Hunziker S, Frei AI, Sutter R (2023) Adverse events related to physical restraint use in intensive care units: a review of the literature. J Intensive Med 4(3):318–325 39035621 10.1016/j.jointm.2023.11.005 PMC11258505

[35] Briskman I, Dubinski R, Barak Y (2010) Treating delirium in a general hospital: a descriptive study of prescribing patterns and outcomes. Int Psychogeriatr 22(2):328–331 19785917 10.1017/S1041610209990986

[36] Yates Z, Lee P, Espat NN, Zagales R, Hernandez N, Amin Q, Ford A, Tweedie C, Elkbuli A (2025) Delirium in critically ill geriatric surgical patients: a systematic review of screening, risk factors, diagnosis, and management. J Trauma Nurs 32(4):169–179 40424371 10.1097/JTN.0000000000000859

