# Supplementary material for: Bridging the gap in delirium management: a European comparison of geriatric practices
Source: Eur Geriatr Med. 2026 Jun 29;17(4):1933–43. doi: 10.1007/s41999-026-01527-6 (PMC13562143; doi:10.1007/s41999-026-01527-6)
Supplement: Supplementary file 1 [file 41999_2026_1527_MOESM2_ESM.pdf]

Survey on diagnostic tools and treatment of delirium across Europe

Dear colleagues,
Thank you very much for participating at this Study.
On the coming pages you will take part at a survey on diagnostic tools and treatment of delirium across Europe.
We ask you for details about your hospital. Mostly it is to allow us to analyse your answers depending on the kind of structures you're working in.
We do not collect any personal Data about you or your patients.
By participating at this Study you accept the terms and conditions.

Thank you for your participation.

The study team

* Indica una domanda obbligatoria

Sezione senza titolo

1) Participant's General Information

1. Country

____

2. City

____

3. Type of structure you are working for *

Contrassegna solo un ovale.

- University hospital
- University-related/affiliated hospital
- Community hospital
- Nursing home
- Rehabilitation center
- Private hospital
- Others

4. Size of the hospital (beds): *

Contrassegna solo un ovale.

- <250
- 250-500
- 500-1000
- >1000

2) Delirium Screening and Diagnosis

A. Geriatric Ward

5. Do you have a geriatric ward? *

Contrassegna solo un ovale.

- Yes
- No *Passa alla domanda 14.*

6. Do you have a screening tool for delirium in the geriatric ward? *

Contrassegna solo un ovale.

- Yes
- No
- I don't know

7. If yes, which one?

Seleziona tutte le voci applicabili.

- [ ] 4AT
- [ ] CAM
- [ ] CAM-ICU
- [ ] DTS
- [ ] ICDSC
- [ ] NU-DESC
- [ ] SQID
- [ ] UB2
- [ ] S-PTD
- [ ] NEWS2
- [ ] RASS
- [ ] RADAR
- [ ] Others

8. Do you have a Protocol for patients with Delirium in the geriatric ward? *

Contrassegna solo un ovale.

- Yes
- No *Passa alla domanda 14.*
- I don't know *Passa alla domanda 14.*

Sezione senza titolo

If yes,

9. Does this pathway include an assessment of frailty?

Contrassegna solo un ovale.

- Yes
- No
- Not sure

10. Does this pathway embed a frailty pathway/bundle/care plan?

Contrassegna solo un ovale.

- Yes
- No
- Not sure

11. Does this pathway have a clear link to a frailty pathway/bundle/care plan?

Contrassegna solo un ovale.

- Yes
- No
- Not sure

12. Does this pathway include referral for follow-up of cognition post an episode delirium?

Contrassegna solo un ovale.

- Yes
- No
- Not sure

13. If this pathway includes follow-up, does the pathway indicate this should be with:

Seleziona tutte le voci applicabili.

- [ ] A delirium service
- [ ] A memory/dementia service
- [ ] A combined dementia/delirium service
- [ ] The treating team from the hospital episode
- [ ] Follow-up recommended but service not specified
- [ ] Others

B. Emergency room

14. Do you have an emergency room? *

Contrassegna solo un ovale.

- ( ) Yes
- ( ) No *Passa alla domanda 25.*

Sezione senza titolo

15. Do you have a screening tool for delirium in the emergency room? *

Contrassegna solo un ovale.

- ( ) Yes
- ( ) No
- ( ) I don’t know

16. If yes, which one?

Seleziona tutte le voci applicabili.

- [ ] 4AT
- [ ] CAM
- [ ] CAM-ICU
- [ ] DTS
- [ ] ICDSC
- [ ] NU-DESC
- [ ] SQID
- [ ] UB2
- [ ] S-PTD
- [ ] NEWS2
- [ ] RASS
- [ ] Others

Sezione senza titolo

17. Do you have diagnosis tool for delirium in the emergency room? *

Contrassegna solo un ovale.

- ( ) Yes
- ( ) No
- ( ) I don't know

18. If yes, which one?

Contrassegna solo un ovale.

- ( ) DSM-IV criteria
- ( ) DSM-V criteria
- ( ) DSM-VI criteria
- ( ) ICD-10 criteria
- ( ) Psychiatric counsel
- ( ) Others

19. Do you have a Protocol for patients with Delirium in the emergency room? *

Contrassegna solo un ovale.

- Yes
- No
- I don't know

If yes,

20. Does this pathway include an assessment of frailty?

Contrassegna solo un ovale.

- Yes
- No
- Not sure

21. Does this pathway embed a frailty pathway/bundle/care plan?

Contrassegna solo un ovale.

- Yes
- No
- Not sure

22. Does this pathway have a clear link to a frailty pathway/bundle/care plan?

Contrassegna solo un ovale.

- Yes
- No
- Not sure

23. Does this pathway include referral for follow-up of cognition post an episode delirium?

Contrassegna solo un ovale.

- Yes
- No
- Not sure

24. If this pathway includes follow-up, does the pathway indicate this should be with:

Seleziona tutte le voci applicabili.

- [ ] A delirium service
- [ ] A memory/dementia service
- [ ] A combined dementia/delirium service
- [ ] The treating team from the hospital episode
- [ ] Follow-up recommended but service not specified
- [ ] Others

C. Intensive care

25. Do you have an intensive care unit? *

Contrassegna solo un ovale.

- Yes
- No *Passa alla domanda 36.*

Sezione senza titolo

26. Do you have a screening tool for delirium in the intensive care unit? *

Contrassegna solo un ovale.

- Yes
- No
- I don't know

27. If yes, which one?

Seleziona tutte le voci applicabili.

- [ ] 4AT
- [ ] CAM
- [ ] CAM-ICU
- [ ] DTS
- [ ] ICDSC
- [ ] NU-DESC
- [ ] SQID
- [ ] UB2
- [ ] S-PTD
- [ ] NEWS2
- [ ] RASS
- [ ] Others

28. Do you have diagnosis tool for delirium in the intensive care unit? *

Contrassegna solo un ovale.

- Yes
- No
- I don't know

29. If yes, which one?

Contrassegna solo un ovale.

- DSM-IV criteria
- DSM-V criteria
- DSM-VI criteria
- ICD-10 criteria
- Psychiatric counsel
- Others

30. Do you have a Protocol for Patients with Delirium on the intensive care unit? *

Contrassegna solo un ovale.

- Yes
- No
- I don't know

If yes,

31. Does this pathway include an assessment of frailty?

Contrassegna solo un ovale.

- Yes
- No
- Not sure

32. Does this pathway embed a frailty pathway/bundle/care plan?

Contrassegna solo un ovale.

- Yes
- No
- Not sure

33. Does this pathway have a clear link to a frailty pathway/bundle/care plan?

Contrassegna solo un ovale.

- Yes
- No
- Not sure

34. Does this pathway include referral for follow-up of cognition post an episode delirium?

Contrassegna solo un ovale.

- Yes
- No
- Not sure

35. If this pathway includes follow-up, does the pathway indicate this should be with:

Seleziona tutte le voci applicabili.

- [ ] A delirium service
- [ ] A memory/dementia service
- [ ] A combined dementia/delirium service
- [ ] The treating team from the hospital episode
- [ ] Follow-up recommended but service not specified
- [ ] Others

D. Medical Ward

36. Do you have medical wards (not geriatric)? *

Contrassegna solo un ovale.

- Yes
- No *Passa alla domanda 47.*

Sezione senza titolo

37. Do you have a screening tool for delirium in the medical ward? *

Contrassegna solo un ovale.

- Yes
- No
- I don't know

38. If yes, which one?

Seleziona tutte le voci applicabili.

- [ ] 4AT
- [ ] CAM
- [ ] CAM-ICU
- [ ] DTS
- [ ] ICDSC
- [ ] NU-DESC
- [ ] SQID
- [ ] UB2
- [ ] S-PTD
- [ ] NEWS2
- [ ] RASS
- [ ] Others

39. Do you have diagnosis tool for delirium in the medical ward? *

Contrassegna solo un ovale.

- Yes
- No
- I don't know

40. If yes, which one?

Contrassegna solo un ovale.

- DSM-IV criteria
- DSM-V criteria
- DSM-VI criteria
- ICD-10 criteria
- Psychiatric counsel
- Others

41. Do you have a Protocol for patients with Delirium in the medical ward? *

Contrassegna solo un ovale.

- Yes
- No
- I don't know

If yes,

42. Does this pathway include an assessment of frailty?

Contrassegna solo un ovale.

- Yes
- No
- Not sure

43. Does this pathway embed a frailty pathway/bundle/care plan?

Contrassegna solo un ovale.

- Yes
- No
- Not sure

44. Does this pathway have a clear link to a frailty pathway/bundle/care plan?

Contrassegna solo un ovale.

- Yes
- No
- Not sure

45. Does this pathway include referral for follow-up of cognition post an episode delirium?

Contrassegna solo un ovale.

- Yes
- No
- Not sure

46. If this pathway includes follow-up, does the pathway indicate this should be with:

Seleziona tutte le voci applicabili.

- [ ] A delirium service
- [ ] A memory/dementia service
- [ ] A combined dementia/delirium service
- [ ] The treating team from the hospital episode
- [ ] Follow-up recommended but service not specified
- [ ] Others

E. Surgery Ward

47. Do you have surgery wards? *

Contrassegna solo un ovale.

- Yes
- No *Passa alla domanda 58.*

Sezione senza titolo

48. Do you have a screening tool for delirium in the surgery ward? *

Contrassegna solo un ovale.

- Yes
- No
- I don't know

49. If yes, which one?

Seleziona tutte le voci applicabili.

- [ ] 4AT
- [ ] CAM
- [ ] CAM-ICU
- [ ] DTS
- [ ] ICDSC
- [ ] NU-DESC
- [ ] SQID
- [ ] UB2
- [ ] S-PTD
- [ ] NEWS2
- [ ] RASS
- [ ] Others

50. Do you have diagnosis tool in the surgery ward? *

Contrassegna solo un ovale.

- Yes
- No
- I don't know

51. If yes, which one?

Contrassegna solo un ovale.

- DSM-IV criteria
- DSM-V criteria
- DSM-VI criteria
- ICD-10 criteria
- Psychiatric counsel
- Others

52. Do you have a Protocol for patients with Delirium in the surgery ward? *

Contrassegna solo un ovale.

- Yes
- No
- I don't know

If yes,

53. Does this pathway include an assessment of frailty?

Contrassegna solo un ovale.

- Yes
- No
- Not sure

54. Does this pathway embed a frailty pathway/bundle/care plan?

Contrassegna solo un ovale.

- Yes
- No
- Not sure

55. Does this pathway have a clear link to a frailty pathway/bundle/care plan?

Contrassegna solo un ovale.

- Yes
- No
- Not sure

56. Does this pathway include referral for follow-up of cognition post an episode delirium?

Contrassegna solo un ovale.

- Yes
- No
- Not sure

57. If this pathway includes follow-up, does the pathway indicate this should be with:

Seleziona tutte le voci applicabili.

- [ ] A delirium service
- [ ] A memory/dementia service
- [ ] A combined dementia/delirium service
- [ ] The treating team from the hospital episode
- [ ] Follow-up recommended but service not specified
- [ ] Others

F. Orthopedic Ward

58. Do you have an orthopedic ward? *

Contrassegna solo un ovale.

- Yes
- No *Passa alla domanda 69.*

Sezione senza titolo

59. Do you have a screening tool for delirium in the orthopedic ward? *

Contrassegna solo un ovale.

- Yes
- No
- I don't know

60. If yes, which one?

Seleziona tutte le voci applicabili.

- [ ] 4AT
- [ ] CAM
- [ ] CAM-ICU
- [ ] DTS
- [ ] ICDSC
- [ ] NU-DESC
- [ ] SQID
- [ ] UB2
- [ ] S-PTD
- [ ] NEWS2
- [ ] RASS
- [ ] Others

61. Do you have diagnosis tool for delirium in the orthopedic ward? *

Contrassegna solo un ovale.

- Yes
- No
- I don't know

62. If yes, which one?

Contrassegna solo un ovale.

- DSM-IV criteria
- DSM-V criteria
- DSM-VI criteria
- ICD-10 criteria
- Psychiatric counsel
- Others

63. Do you have a Protocol for Patients with Delirium on orthopedic ward? *

Contrassegna solo un ovale.

- Yes
- No
- I don't know

If yes,

64. Does this pathway include an assessment of frailty?

Contrassegna solo un ovale.

- Yes
- No
- Not sure

65. Does this pathway embed a frailty pathway/bundle/care plan?

Contrassegna solo un ovale.

- Yes
- No
- Not sure

66. Does this pathway have a clear link to a frailty pathway/bundle/care plan?

Contrassegna solo un ovale.

- Yes
- No
- Not sure

67. Does this pathway include referral for follow-up of cognition post an episode delirium?

Contrassegna solo un ovale.

- Yes
- No
- Not sure

68. If this pathway includes follow-up, does the pathway indicate this should be with:

Seleziona tutte le voci applicabili.

- [ ] A delirium service
- [ ] A memory/dementia service
- [ ] A combined dementia/delirium service
- [ ] The treating team from the hospital episode
- [ ] Follow-up recommended but service not specified
- [ ] Others

G. Rehabilitation ward

69. Do you have a rehabilitation ward in your setting? *

Contrassegna solo un ovale.

- Yes
- No *Passa alla domanda 80.*

Sezione senza titolo

70. Do you have a screening tool for delirium in the rehabilitation ward? *

Contrassegna solo un ovale.

- Yes
- No
- I don't know

71. If yes, which one?

Seleziona tutte le voci applicabili.

- [ ] 4AT
- [ ] CAM
- [ ] CAM-ICU
- [ ] DTS
- [ ] ICDSC
- [ ] NU-DESC
- [ ] SQID
- [ ] UB2
- [ ] S-PTD
- [ ] NEWS2
- [ ] RASS
- [ ] Others

72. Do you have diagnosis tool for delirium in the rehabilitation ward? *

Contrassegna solo un ovale.

- ( ) Yes
- ( ) No
- ( ) I don't know

73. If yes, which one?

Contrassegna solo un ovale.

- ( ) DSM-IV criteria
- ( ) DSM-V criteria
- ( ) DSM-VI criteria
- ( ) ICD-10 criteria
- ( ) Psychiatric counsel
- ( ) Others

74. Do you have a Protocol for patients with Delirium on rehabilitation ward? *

Contrassegna solo un ovale.

- Yes
- No
- I don't know

If yes,

75. Does this pathway include an assessment of frailty?

Contrassegna solo un ovale.

- Yes
- No
- Not sure

76. Does this pathway embed a frailty pathway/bundle/care plan?

Contrassegna solo un ovale.

- Yes
- No
- Not sure

77. Does this pathway have a clear link to a frailty pathway/bundle/care plan?

Contrassegna solo un ovale.

- Yes
- No
- Not sure

78. Does this pathway include referral for follow-up of cognition post an episode delirium?

Contrassegna solo un ovale.

- Yes
- No
- Not sure

79. If this pathway includes follow-up, does the pathway indicate this should be with:

Seleziona tutte le voci applicabili.

- [ ] A delirium service
- [ ] A memory/dementia service
- [ ] A combined dementia/delirium service
- [ ] The treating team from the hospital episode
- [ ] Follow-up recommended but service not specified
- [ ] Others

H. Nursing Home

80. Do you have a nursing home (long-term residential) beds in your setting? *

Contrassegna solo un ovale.

- Yes
- No *Passa alla domanda 91.*

Sezione senza titolo

81. Do you have a screening tool for delirium in the nursing home? *

Contrassegna solo un ovale.

- Yes
- No
- I don't know

82. If yes, which one?

Seleziona tutte le voci applicabili.

- [ ] 4AT
- [ ] CAM
- [ ] CAM-ICU
- [ ] DTS
- [ ] ICDSC
- [ ] NU-DESC
- [ ] SQID
- [ ] UB2
- [ ] S-PTD
- [ ] NEWS2
- [ ] RASS
- [ ] Others

83. Do you have diagnosis tool for delirium in the nursing home? *

Contrassegna solo un ovale.

- Yes
- No
- I don't know

84. If yes, which one?

Contrassegna solo un ovale.

- DSM-IV criteria
- DSM-V criteria
- DSM-VI criteria
- ICD-10 criteria
- Psychiatric counsel
- Others

85. Do you have a Protocol for patients with Delirium in the nursing home? *

Contrassegna solo un ovale.

- Yes
- No
- I don't know

If yes,

86. Does this pathway include an assessment of frailty?

Contrassegna solo un ovale.

- Yes
- No
- Not sure

87. Does this pathway embed a frailty pathway/bundle/care plan?

Contrassegna solo un ovale.

- Yes
- No
- Not sure

88. Does this pathway have a clear link to a frailty pathway/bundle/care plan?

Contrassegna solo un ovale.

- Yes
- No
- Not sure

89. Does this pathway include referral for follow-up of cognition post an episode delirium? *

Contrassegna solo un ovale.

- Yes
- No
- Not sure

90. If this pathway includes follow-up, does the pathway indicate this should be with:

Seleziona tutte le voci applicabili.

- [ ] A delirium service
- [ ] A memory/dementia service
- [ ] A combined dementia/delirium service
- [ ] The treating team from the hospital episode
- [ ] Follow-up recommended but service not specified
- [ ] Others

3) Delirium treatment

91. Which type of delirium do you treat? *

Seleziona tutte le voci applicabili.

- [ ] Hyperactive
- [ ] Hypoactive
- [ ] Mixed
- [ ] All

3.1 Delirium treatment with NON-pharmacological interventions

Do delirious patients of your unit/ward receive the following NON pharmacological interventions?

92. Mobilization (sitting on the edge of bed, transfer bed – chair) *

Contrassegna solo un ovale.

- Yes
- No *Passa alla domanda 94.*
- I don't know *Passa alla domanda 94.*

Sezione senza titolo

93. In which percentage? *

Contrassegna solo un ovale.

- < 25
- 25-50
- 50-75
- >75

Sezione senza titolo

94. Support to Walk more (eg extra staff are provided to support this)? *

Contrassegna solo un ovale.

- Yes
- No *Passa alla domanda 96.*
- I don't know *Passa alla domanda 96.*

Sezione senza titolo

95. In which percentage? *

Contrassegna solo un ovale.

- < 25
- 25-50
- 50-75
- >75

Sezione senza titolo

96. Pain assessment? *

Contrassegna solo un ovale.

- Yes
- No *Passa alla domanda 98.*
- I don't know *Passa alla domanda 98.*

Sezione senza titolo

97. In which percentage? *

Contrassegna solo un ovale.

- < 25
- 25-50
- 50-75
- >75

Sezione senza titolo

98. Pain management (if pain detected) with non pharmacological interventions (can be additional to medications)? *

Contrassegna solo un ovale.

- Yes
- No *Passa alla domanda 100.*
- I don't know *Passa alla domanda 100.*

Sezione senza titolo

99. In which percentage? *

Contrassegna solo un ovale.

- < 25
- 25-50
- 50-75
- >75

Sezione senza titolo

100. Physical restraints (e.g. bed rails, wrist bands and others) *

Contrassegna solo un ovale.

- Yes
- No *Passa alla domanda 102.*
- I don't know *Passa alla domanda 102.*

Sezione senza titolo

101. In which percentage? *

Contrassegna solo un ovale.

- < 25
- 25-50
- 50-75
- >75

Sezione senza titolo

102. Write the different types of restraints: *

Seleziona tutte le voci applicabili.

- [ ] Only bed rails
- [ ] Bed rails and wrist bands
- [ ] Bed rails and ankle bands
- [ ] Bed rails and wrist and ankle bands
- [ ] Bed rails and belt

Sezione senza titolo

103. Care in reactivating day and night cycles *

Contrassegna solo un ovale.

- Yes
- No *Passa alla domanda 105.*
- I don't know *Passa alla domanda 105.*

Sezione senza titolo

104. In which percentage? *

Contrassegna solo un ovale.

- < 25
- 25-50
- 50-75
- >75

Sezione senza titolo

105. Support to increase fluids intake (e.g. extra staff support, fluid charts) *

Contrassegna solo un ovale.

- Yes
- No *Passa alla domanda 107.*
- I don't know *Passa alla domanda 107.*

Sezione senza titolo

106. In which percentage? *

Contrassegna solo un ovale.

- < 25
- 25-50
- 50-75
- >75

Sezione senza titolo

107. Provision of vision and hearing and mobility aids *

Contrassegna solo un ovale.

- Yes
- No *Passa alla domanda 109.*
- I don't know *Passa alla domanda 109.*

Sezione senza titolo

108. In which percentage? *

Contrassegna solo un ovale.

- < 25
- 25-50
- 50-75
- >75

Sezione senza titolo

109. Cognitive stimulation with ADL (e.g. provision of newspapers, music, other) *

Contrassegna solo un ovale.

- Yes
- No *Passa alla domanda 111.*
- I don't know *Passa alla domanda 111.*

Sezione senza titolo

110. In which percentage? *

Contrassegna solo un ovale.

- < 25
- 25-50
- 50-75
- >75

Sezione senza titolo

111. Verbal reorientation *

Contrassegna solo un ovale.

- Yes
- No *Passa alla domanda 113.*
- I don't know *Passa alla domanda 113.*

Sezione senza titolo

112. In which percentage? *

Contrassegna solo un ovale.

- < 25
- 25-50
- 50-75
- >75

Sezione senza titolo

113. Open or liberal visiting times for families (daytime, permission to caregiver to stay with patient and let them do ADL) *

Contrassegna solo un ovale.

- Yes
- No *Passa alla domanda 115.*
- I don't know *Passa alla domanda 115.*

Sezione senza titolo

114. In which percentage? *

Contrassegna solo un ovale.

- < 25
- 25-50
- 50-75
- >75

Sezione senza titolo

115. Caregiver education for proactive and informed caregiving *

Contrassegna solo un ovale.

- Yes
- No *Passa alla domanda 117.*
- I don't know *Passa alla domanda 117.*

Sezione senza titolo

116. In which percentage? *

Contrassegna solo un ovale.

- < 25
- 25-50
- 50-75
- >75

Sezione senza titolo

117. Transferring to specific delirium rooms? *

Contrassegna solo un ovale.

- Yes
- No *Passa alla domanda 119.*
- I don't know *Passa alla domanda 119.*

Sezione senza titolo

118. In which percentage? *

Contrassegna solo un ovale.

- < 25
- 25-50
- 50-75
- >75

Sezione senza titolo

119. Non-disturbed sleep (i.e., reduction of noise and light) *

Contrassegna solo un ovale.

- Yes
- No *Passa alla domanda 121.*
- I don't know *Passa alla domanda 121.*

Sezione senza titolo

120. In which percentage? *

Contrassegna solo un ovale.

- < 25
- 25-50
- 50-75
- >75

Sezione senza titolo

121. Avoidance of bladder tubes/catheters if not necessary? *

Contrassegna solo un ovale.

- Yes
- No *Passa alla domanda 123.*
- I don't know *Passa alla domanda 123.*

Sezione senza titolo

122. In which percentage? *

Contrassegna solo un ovale.

- < 25
- 25-50
- 50-75
- >75

Sezione senza titolo

123. Sharing or communicating patient information about delirium with other staff members? *

Contrassegna solo un ovale.

- Yes
- No *Passa alla domanda 125.*
- I don't know *Passa alla domanda 125.*

Sezione senza titolo

124. In which percentage? *

Contrassegna solo un ovale.

- < 25
- 25-50
- 50-75
- >75

Sezione senza titolo

125. Ground-leveled beds? *

Contrassegna solo un ovale.

- Yes
- No *Passa alla domanda 127.*
- I don't know *Passa alla domanda 127.*

Sezione senza titolo

126. In which percentage? *

Contrassegna solo un ovale.

- < 25
- 25-50
- 50-75
- >75

Sezione senza titolo

127. Activities in patient groups (e.g. singing, eating, doing exercises together)? *

Contrassegna solo un ovale.

- Yes
- No *Passa alla domanda 129.*
- I don't know *Passa alla domanda 129.*

Sezione senza titolo

128. In which percentage? *

Contrassegna solo un ovale.

- < 25
- 25-50
- 50-75
- >75

Sezione senza titolo

129. Animal assisted therapy? *

Contrassegna solo un ovale.

- Yes
- No *Passa alla domanda 131.*
- I don't know *Passa alla domanda 131.*

Sezione senza titolo

130. In which percentage? *

Contrassegna solo un ovale.

- < 25
- 25-50
- 50-75
- >75

Sezione senza titolo

131. Going “outside” the unit/ward, eg hospital hall, garden, sunlight? *

Contrassegna solo un ovale.

- Yes
- No *Passa alla domanda 133.*
- I don't know *Passa alla domanda 133.*

Sezione senza titolo

132. In which percentage? *

Contrassegna solo un ovale.

- < 25
- 25-50
- 50-75
- >75

Sezione senza titolo

133. Reduction of potentially delirogenous drugs? *

Contrassegna solo un ovale.

- Yes
- No *Passa alla domanda 135.*
- I don't know *Passa alla domanda 135.*

Sezione senza titolo

134. In which percentage? *

Contrassegna solo un ovale.

- < 25
- 25-50
- 50-75
- >75

Sezione senza titolo

135. Which professionals predominantly provide the non-pharmacological treatments for people with delirium? (max 3 professions) *

Seleziona tutte le voci applicabili.

- Nurse
- Physician
- Physiotherapist
- Nurse assistant
- Occupational therapist
- Speech therapist
- Psychologist/neuropshycologist
- Social Worker
- Other

136. How satisfied do you feel with the nonpharmacological treatments for people with delirium in your unit? *

Contrassegna solo un ovale.

	1	2	3	4	5	
not ¿						completely satisfied

3.2 Delirium Pharmacological treatment

137. Which type of delirium do you treat? *

Seleziona tutte le voci applicabili.

- [ ] Hyperkinetic
- [ ] Hypokinetic
- [ ] Mixed
- [ ] All types

138. Do delirious patients of your unit/ward receive pharmacological interventions? *

Contrassegna solo un ovale.

- ( ) Yes
- ( ) No
- ( ) I don't know

139. In which percentage? *

Contrassegna solo un ovale.

- ( ) < 25
- ( ) 25-50
- ( ) 50-75
- ( ) >75

140. Which of the following medications are used on your ward? *

Seleziona tutte le voci applicabili.

- [ ] Haloperidol
- [ ] Clonidine
- [ ] Promazine
- [ ] Trazodone
- [ ] Melperone
- [ ] Risperidone
- [ ] Olanzapine
- [ ] Lorazepam
- [ ] Dexmedetomidine
- [ ] Diazepam
- [ ] Quetiapine
- [ ] Midazolam
- [ ] Distraneurin
- [ ] Melatonin
- [ ] Beta-blocker
- [ ] Pipamperone
- [ ] Levodopa
- [ ] Phenobarbital
- [ ] Do not know
- [ ] Other (please specify)

141. Do you have chosen Haloperidol? *

Contrassegna solo un ovale.

- ( ) Yes
- ( ) No *Passa alla domanda 146.*

142. Mean dosage for administration (mg) *

____

143. Mean number of administrations per day *

____

144. Total dosage per day (mg) *

____

145. Type of administration: *

Seleziona tutte le voci applicabili.

- [ ] Oral
- [ ] Intramuscular
- [ ] Intravenous

146. Do you have chosen Clonidine? *

Contrassegna solo un ovale.

- ( ) Yes
- ( ) No *Passa alla domanda 151.*

Sezione senza titolo

147. Mean dosage for administration (mg) *

____

148. Mean number of administrations per day *

____

149. Total dosage per day (mg) *

____

150. Type of administration: *

Seleziona tutte le voci applicabili.

- [ ] Oral
- [ ] Intramuscular
- [ ] Intravenous

151. Do you have chosen Promazine? *

Contrassegna solo un ovale.

- ( ) Yes
- ( ) No *Passa alla domanda 156.*

Sezione senza titolo

152. Mean dosage for administration (mg) *

____

153. Mean number of administrations per day *

____

154. Total dosage per day (mg) *

____

155. Type of administration: *

Seleziona tutte le voci applicabili.

- [ ] Oral
- [ ] Intramuscular
- [ ] Intravenous

Sezione senza titolo

156. Do you have chosen Trazodone? *

Contrassegna solo un ovale.

- Yes
- No *Passa alla domanda 161.*

Sezione senza titolo

157. Mean dosage for administration (mg) *

____

158. Mean number of administrations per day *

____

159. Total dosage per day (mg) *

____

160. Type of administration: *

Seleziona tutte le voci applicabili.

- [ ] Oral
- [ ] Intramuscular
- [ ] Intravenous

Sezione senza titolo

161. Do you have chosen Melperone? *

Contrassegna solo un ovale.

- Yes
- No *Passa alla domanda 166.*

Sezione senza titolo

162. Mean dosage for administration (mg) *

163. Mean number of administrations per day *

164. Total dosage per day (mg) *

165. Type of administration: *

Seleziona tutte le voci applicabili.

- [ ] Oral
- [ ] Intramuscular
- [ ] Intravenous

Sezione senza titolo

166. Do you have chosen Risperidone? *

Contrassegna solo un ovale.

- ( ) Yes
- ( ) No *Passa alla domanda 171.*

Sezione senza titolo

167. Mean dosage for administration (mg) *

168. Mean number of administrations per day *

169. Total dosage per day (mg) *

170. Type of administration: *

Seleziona tutte le voci applicabili.

- [ ] Oral
- [ ] Intramuscular
- [ ] Intravenous

Sezione senza titolo

171. Do you have chosen Olanzapine? *

Contrassegna solo un ovale.

- ( ) Yes
- ( ) No *Passa alla domanda 176.*

Sezione senza titolo

172. Mean dosage for administration (mg) *

173. Mean number of administrations per day *

174. Total dosage per day (mg) *

175. Type of administration: *

Seleziona tutte le voci applicabili.

- [ ] Oral
- [ ] Intramuscular
- [ ] Intravenous

Sezione senza titolo

176. Do you have chosen Lorazepam? *

Contrassegna solo un ovale.

- ( ) Yes
- ( ) No *Passa alla domanda 181.*

Sezione senza titolo

177. Mean dosage for administration (mg) *

____

178. Mean number of administrations per day *

____

179. Total dosage per day (mg) *

____

180. Type of administration: *

Seleziona tutte le voci applicabili.

- [ ] Oral
- [ ] Intramuscular
- [ ] Intravenous

Sezione senza titolo

181. Do you have chosen Dexmedetomidine? *

Contrassegna solo un ovale.

- Yes
- No *Passa alla domanda 186.*

Sezione senza titolo

182. Mean dosage for administration (mg) *

183. Mean number of administrations per day *

184. Total dosage per day (mg) *

185. Type of administration: *

Seleziona tutte le voci applicabili.

- [ ] Oral
- [ ] Intramuscular
- [ ] Intravenous

Sezione senza titolo

186. Do you have chosen Diazepam? *

Contrassegna solo un ovale.

- Yes
- No *Passa alla domanda 191.*

Sezione senza titolo

187. Mean dosage for administration (mg) *

188. Mean number of administrations per day *

189. Total dosage per day (mg) *

190. Type of administration: *

Seleziona tutte le voci applicabili.

- [ ] Oral
- [ ] Intramuscular
- [ ] Intravenous

Sezione senza titolo

191. Do you have chosen Quetiapine? *

Contrassegna solo un ovale.

- Yes
- No *Passa alla domanda 196.*

Sezione senza titolo

192. Mean dosage for administration (mg) *

193. Mean number of administrations per day *

194. Total dosage per day (mg) *

195. Type of administration: *

Seleziona tutte le voci applicabili.

- [ ] Oral
- [ ] Intramuscular
- [ ] Intravenous

Sezione senza titolo

196. Do you have chosen Midazolam? *

Contrassegna solo un ovale.

- ( ) Yes
- ( ) No *Passa alla domanda 201.*

Sezione senza titolo

197. Mean dosage for administration (mg) *

198. Mean number of administrations per day *

199. Total dosage per day (mg) *

200. Type of administration: *

Seleziona tutte le voci applicabili.

- [ ] Oral
- [ ] Intramuscular
- [ ] Intravenous

Sezione senza titolo

201. Do you have chosen Distraneurin? *

Contrassegna solo un ovale.

- ( ) Yes
- ( ) No *Passa alla domanda 206.*

Sezione senza titolo

202. Mean dosage for administration (mg) *

203. Mean number of administrations per day *

204. Total dosage per day (mg) *

205. Type of administration: *

Seleziona tutte le voci applicabili.

- [ ] Oral
- [ ] Intramuscular
- [ ] Intravenous

Sezione senza titolo

206. Do you have chosen Melatonin? *

Contrassegna solo un ovale.

- ( ) Yes
- ( ) No *Passa alla domanda 211.*

Sezione senza titolo

207. Mean dosage for administration (mg) *

____

208. Mean number of administrations per day *

____

209. Total dosage per day (mg) *

____

210. Type of administration: *

Seleziona tutte le voci applicabili.

- [ ] Oral
- [ ] Intramuscular
- [ ] Intravenous

Sezione senza titolo

211. Do you have chosen Beta-Blocker? *

Contrassegna solo un ovale.

- Yes
- No *Passa alla domanda 216.*

Sezione senza titolo

212. Mean dosage for administration (mg) *

213. Mean number of administrations per day *

214. Total dosage per day (mg) *

215. Type of administration: *

Seleziona tutte le voci applicabili.

- [ ] Oral
- [ ] Intramuscular
- [ ] Intravenous

Sezione senza titolo

216. Do you have chosen Pipamperone? *

Contrassegna solo un ovale.

- Yes
- No *Passa alla domanda 221.*

Sezione senza titolo

217. Mean dosage for administration (mg) *

218. Mean number of administrations per day *

219. Total dosage per day (mg) *

220. Type of administration: *

Seleziona tutte le voci applicabili.

- [ ] Oral
- [ ] Intramuscular
- [ ] Intravenous

Sezione senza titolo

221. Do you have chosen Levodopa? *

Contrassegna solo un ovale.

- Yes
- No *Passa alla domanda 226.*

Sezione senza titolo

222. Mean dosage for administration (mg) *

223. Mean number of administrations per day *

224. Total dosage per day (mg) *

225. Type of administration: *

Seleziona tutte le voci applicabili.

- [ ] Oral
- [ ] Intramuscular
- [ ] Intravenous

Sezione senza titolo

226. Do you have chosen Phenobarbital? *

Contrassegna solo un ovale.

- ( ) Yes
- ( ) No

Sezione senza titolo

227. Mean dosage for administration (mg) *

228. Mean number of administrations per day *

229. Total dosage per day (mg) *

230. Type of administration: *

Seleziona tutte le voci applicabili.

- [ ] Oral
- [ ] Intramuscular
- [ ] Intravenous

Questi contenuti non sono creati né avallati da Google.

Google Moduli
