# Supplementary material for: Bridging the gap in delirium management: a European comparison of geriatric practices
Source: Eur Geriatr Med. 2026 Jun 29;17(4):1933–43. doi: 10.1007/s41999-026-01527-6 (PMC13562143; doi:10.1007/s41999-026-01527-6)
Supplement: Supplementary file 2 [file 41999_2026_1527_MOESM1_ESM.docx]

**Supplementary Table 1**

| **Variables** | **Less-established GM (N=41)** | **Well-established GM (N=155)** | **P-value** |
| --- | --- | --- | --- |
| **Presence of a Rehabilitation ward** | 20 (48.8) | 73 (47.1) | 0.848 |
| Screening tool for delirium | 6 (14.6) | 36 (23.2) | 0.442 |
| Protocol for patients with delirium | 5 (12.2) | 28 (18.1) | 0.337 |
| Pathway including frailty assessment | 4 (9.8) | 22 (14.2) | 0.580 |
| Pathway including frailty bundle care plan | 5 (12.2) | 18 (11.6) | 0.303 |
| Clear link with frailty bundle care plan | 4 (9.8) | 17 (11) | 0.576 |
| Referral for follow-up of cognition | 6 (14.6) | 19 (12.3) | 0.579 |
| **Presence of a Long-term residential unit** | 7 (17.1) | 25 (16.1) | 0.884 |
| Screening tool for delirium | 3 (7.3) | 11 (7.1) | 0.979 |
| Protocol for patients with delirium | 1 (2.4) | 6 (3.9) | 0.948 |
| Pathway including frailty assessment | 1 (2.4) | 2 (1.3) | 0.828 |
| Pathway including frailty bundle care plan | 1 (2.4) | 2 (1.3) | 0.741 |
| Clear link with frailty bundle care plan | 1 (2.4) | 2 (1.3) | 0.741 |
| Referral for follow-up of cognition | 1 (2.4) | 6 (3.9) | 0.511 |
